# Case Report: Multimodal Imaging Features of Pedunculated Liver Masses in Seven Dogs

**DOI:** 10.3389/fvets.2020.581922

**Published:** 2020-11-24

**Authors:** Jaeeun Ko, Jeongyeon Hwang, Hakyoung Yoon, Kidong Eom, Jaehwan Kim

**Affiliations:** ^1^Department of Veterinary Medical Imaging, College of Veterinary Medicine, Konkuk University, Seoul, South Korea; ^2^Department of Veterinary Medical Imaging, College of Veterinary Medicine, Jeonbuk National University, Jeonju, South Korea

**Keywords:** accessory liver, computed tomography, ultrasonography, dog, pedicle, pedunculated liver mass

## Abstract

This study describes the multimodal imaging characteristics of pedunculated liver masses in seven dogs [Cocker Spaniel (*n* = 2), Maltese (*n* = 1), Shih-Tzu (*n* = 2), and Schnauzer (*n* = 2)]. These masses are anatomic variants of hepatic masses in which the center of the mass lies outside the liver contour. Prior to referral, only one dog had been diagnosed with a hepatic mass, four had been diagnosed with mid-abdominal masses of unknown origin, and two had been misdiagnosed with splenic head and pancreatic masses. Using radiographs, the mass locations were classified as cranioventral (*n* = 3), mid-abdominal (*n* = 2), or craniodorsal (*n* = 2). The gastric axis was deviated in various directions in four cases. Based on computed tomography (CT) findings, the masses were noted to originate from every liver lobe (two from the left lateral lobe) and to possess parenchymal (*n* = 6) or vascular (*n* = 1) pedicles. The histopathological results showed that three masses were benign [hepatic adenoma (*n* = 1) and nodular hyperplasia (*n* = 2)] and four were malignant [hepatocellular carcinoma (*n* = 3) and cholangiocarcinoma (*n* = 1)]. For three dogs, triple-phase CT maximum intensity projection images in the arterial phase clearly showed that the masses were connected to the hepatic artery. We propose that a pedunculated liver mass should be considered as a differential diagnosis when a mass is located in the mid-abdomen, even if it is separated from the liver and with the gastric axis deviated in various directions. We consider CT imaging to be a useful tool for diagnosis, evaluation, and surgical planning in dogs with a pedunculated liver mass.

## Introduction

A pedunculated or exophytic liver mass is defined as a protruding mass that is connected to the liver by a pedicle and has a center that lies beyond the contour of the liver (1, 2). The differential diagnoses for this type of mass are the same as those for other benign (e.g., hepatic cyst, focal nodular hyperplasia, hepatocellular adenoma, etc.) or malignant (e.g., hepatocellular adenocarcinoma, cholangiocarcinoma, etc.) hepatic masses. This type of mass is rare in animals as well as in humans (3); however, a careful evaluation of masses that initially appear to arise in the mid-abdomen is important since a pedunculated hepatic mass may be mistaken for one originating from a different organ, such as the stomach, small intestine, pancreas, and mesenteric lymph node, while those arising more dorsally may be mistaken for the adrenal gland (4–6). Various types of pedunculated liver masses, such as hepatocellular carcinoma (HCC), cholangiocarcinoma, adenoma, hemangioma, and various cystic hepatic masses, have been reported in humans, and their respective computed tomographic (CT) imaging features have been established (1, 2). Conversely, although hepatic masses are relatively common in the veterinary setting, pedunculated liver masses have not been previously reported in the literature (7, 8). Thus, the aim of this study was to describe the radiographic, ultrasonographic, and CT features of pedunculated liver masses in dogs.

## Materials and Methods

From February 2014 to December 2018, the medical record databases of two different animal referral hospitals were searched for dogs with pedunculated liver masses. For inclusion in the study, the dogs were required to have complete records that included information regarding their breed, age, sex, chief complaint, serum biochemistry results, including alanine aminotransferase (ALT), aspartate aminotransferase (AST), alkaline phosphatase (ALP), and gamma glutamyltransferase (GGT) levels (Catalyst Dx, IDEXX Laboratories, Inc., Westbrook, Maine, USA) and the results of histopathological or fine-needle aspiration of the mass. Findings of at least two imaging modalities, including radiography, were also required. The owners of the enrolled dogs were informed about the study and consented to the use of the medical records of their dogs.

Abdominal radiography with right lateral and ventrodorsal projections (Titan 2000M; Comed Medical Systems Co., Ltd., Seoul, Korea) and image analysis were routinely performed. Lateral radiographs were used to categorize the locations of the masses as previously described (9). Briefly, the masses were classified as mid-abdominal if either small or large intestinal loops were displaced both cranially and caudally to the mass. Deviation of the gastric axis, defined as the hypothetical line connecting the gastric fundus and the pylorus, on lateral radiographs was also assessed. Abdominal ultrasonography (US) was routinely performed (Prosound F75 and Prosound a6; Aloka, Tokyo, Japan) using linear-array (10–13 MHz) and curvilinear-array (6–8 MHz) probes to determine the echogenicity, size, and vascular distribution of the abdominal masses. Vascular supply to the mass was investigated whenever possible. The probable origin of each mass was determined based on the abovementioned imaging findings.

The same anesthetic and CT protocols were used at both institutions involved in this study. For CT scans, anesthesia was induced by the intravenous administration of 6 mg/kg of propofol (Provive; Myungmoon Pharmaceutical Co., Seoul, Korea) and maintained using 1.5 % isoflurane (Forane solution; Choongwae Pharma Corporation, Seoul, Korea) in 100% oxygen administered *via* endotracheal intubation. CT scans were acquired from all dogs in ventral recumbency using a four-multi-detector (Lightspeed; GE Medical Systems, Milwaukee, WI, USA) or a 16-multi-detector (Brivo CT 385; GE Medical Systems) system. The imaging parameters were as follows: 120 kVp, 200 mA, and 1.25-mm slice thickness in helical scan mode. Using a power injector (CT power injector, GE Medical Systems), 600 mg iodine/kg iohexol (Omnihexol 300; Korea United Pharmaceutical, Seoul, Korea) was injected into the cephalic vein at a rate of 1.5 ml/s. Triple-phase CT images (arterial, portal, and equilibrium phases) were obtained using the bolus tracking techniques in dogs with a suspected hepatic mass based on radiographic or ultrasonographic examinations. The threshold for arterial phase imaging was set to 300 Hounsfield units in the descending aorta at the level of the dome of the diaphragm. After 20 s and another 40 s of arterial phase imaging, portal and equilibrium phase scan images were acquired. The scanning range for the pre-contrast and equilibrium phases was set from head to feet; for the arterial and the portal phases, it was set according to the size and the location of the mass. All acquired images were reviewed by experienced radiologists using the commercially available OsiriX 9.0 software (Pixmeo, Geneva, Switzerland) and the soft tissue window (window level: 60, window width: 400) or lung window (window level: −400, window width: 1,500) based on the region of interest.

Based on the CT images, the maximum dimensions (length, width, and height in centimeters) of each mass were determined. The minimum diameter of the pedicles was measured in three orthogonal planes. In addition, we also assessed whether a liver lobe was connected to the mass. A mass was confirmed to have a hepatic origin if it was connected to the hepatic artery, portal vein, or hepatic vein. Hepatic pedicles in humans are classified as one of three types based on their attachment to the liver: sessile lobe, pedunculated lobe, or ectopic lobe (10). As there are no detailed reports on hepatic pedicles in canine species, the masses were simply categorized as parenchymal, if they included hepatic parenchyma, or vascular, if they consisted of only blood vessels and bile ducts without hepatic parenchyma. In some dogs, the hepatic vasculature was assessed using maximum intensity projection (MIP) to further clarify the association between the mass and the liver. Thoracic and abdominal lymph nodes and lungs were also evaluated.

## Results

Seven dogs [Cocker Spaniel (*n* = 2), Maltese (*n* = 1), Shih-Tzu (*n* = 2), and Schnauzer (*n* = 2); three castrated males and four spayed females] met the inclusion criteria. All relevant information and chief complaints are available as Supplementary Material 1. The mean age ± standard deviation was 12 ± 1.9 years (range, 9–14 years), and the mean body weight was 7.1 ± 2.6 kg (range, 3.7–11.3 kg). Only one out of the seven dogs had been correctly diagnosed with a hepatic mass prior to referral. Two other dogs had been misdiagnosed as having either a splenic head mass (dog 6) or a pancreatic mass (dog 7) prior to referral. Three dogs (dogs 1, 2, and 3) had been diagnosed with an abdominal mass of unknown origin prior to referral and had been referred for CT scans. The mass of the last dog (dog 5) was incidentally detected during treatment for a skin disease.

The serum biochemistry profiles showed elevated ALT, AST, ALP, and GGT levels in all dogs, except in dogs 6 and 7, who had normal hepatic enzyme profiles.

**Figure 1:**
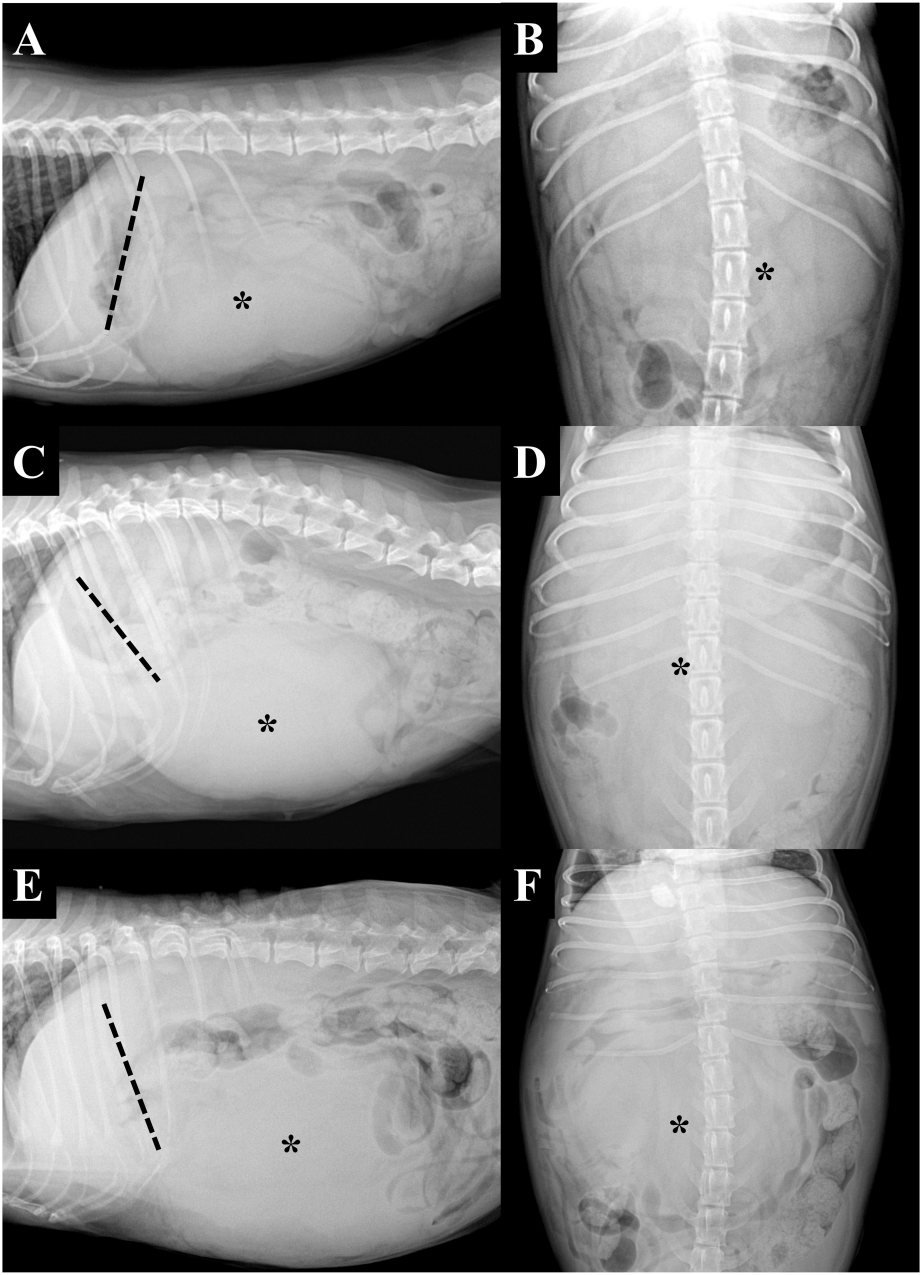
Right lateral **(A,C,E)** and ventrodorsal **(B,D,F)** projection radiographs of three dogs with pedunculated liver masses (asterisks). The tip of the liver margin is clearly visible and can be observed to be separate from the mass in all instances. The masses were located in the cranioventral **(A)** and mid-abdominal **(C,E)** regions. Note that the gastric axis (dashed lines) can be deviated in various directions, including the cranial **(A)** and caudodorsal **(C)** direction, or be in the normal position **(E)**. **(A,B)** Dog 2, **(C,D)** dog 3, and **(E,F)** dog 4.

The imaging findings from the present study are summarized in Table 1. Based on the radiographs (Figure 1), the mass locations were classified as cranioventral (*n* = 3), mid-abdominal (*n* = 2), or craniodorsal (*n* = 2). The gastric axis was in normal position in three dogs (dogs 4, 6, and 7); in the other dogs, it was deviated either cranially (*n* = 2), caudodorsally (*n* = 1), or dorsally (*n* = 1).

**Table 1:** Summary of the radiographic, ultrasonographic, computed tomographic, and histopathologic results in seven dogs with a pedunculated liver mass.

**Case**	**Radiography**		**Ultrasonography**		**Computed tomography**					**Final diagnosis**
	**Mass location**	**GA deviation**	**Echogenicity/texture of the mass^a^**	**Other findings**	**Origin**	**Pedicle type**	**Pedicle size (cm)**	**Mass size (cm)**	**Other findings**	
1	Cranioventral	Dorsal	–	–	LLL	Parenchymal	2.1	10 × 5 × 10	Lymphadenopathy (hepatic)	Hepatic adenoma^b^
2	Cranioventral	Cranial	Multifocally cystic echotexture hypoechoic	Lymphadenopathy (hepatic, splenic)	QLL	Vascular	0.5	6 × 5 × 5	Lymphadenopathy (hepatic, splenic)	HCC
3	Mid-abdomen	Caudodorsal	–	–	LLL	Parenchymal	1.7	10 × 6 × 12	Lymphadenopathy (hepatic) Peri-tumoral peritonitis	Cholangiocarcinoma
4	Mid-abdomen	Normal	Fine echotexture heterogeneous, predominantly hypoechoic	Peri-tumoral peritonitis ascites	LML	Parenchymal	1.3	10 × 11 × 11	Lymphadenopathy (sternal, pancreaticoduodenal) Peri-tumoral peritonitis Ascites	HCC
5	Cranioventral	Cranial	Fine echotexture hyperechoic with radiating hypoechoic stripe	Peri-tumoral peritonitis	RML	Parenchymal	1.3	6 × 5 × 5	Lymphadenopathy (hepatic) peri-tumoral peritonitis	HCC
6	Craniodorsal	Normal	–	–	RLL	Parenchymal	1.9	4 × 5 × 4	-	NH^b^
7	Craniodorsal	Normal	Fine echotexture heterogeneous, predominantly hyperechoic	–	CLL	Parenchymal	1.3	2 × 2 × 3	-	NH

*Other findings are focused on the mass-associated changes*.

*GA, gastric axis; LLL, left lateral liver lobe; LML, left medial lobe; QLL, quadrate liver lobe; RML, right medial liver lobe; RLL, right lateral liver lobe; CLL, caudate liver lobe; HCC, hepatocellular carcinoma; NH, nodular hyperplasia; mass size, length × width × height*.

a *Echogenicity compared to normal hepatic parenchyma*.

b *Diagnosed based on fine-needle aspiration*.

**Figure 2:**
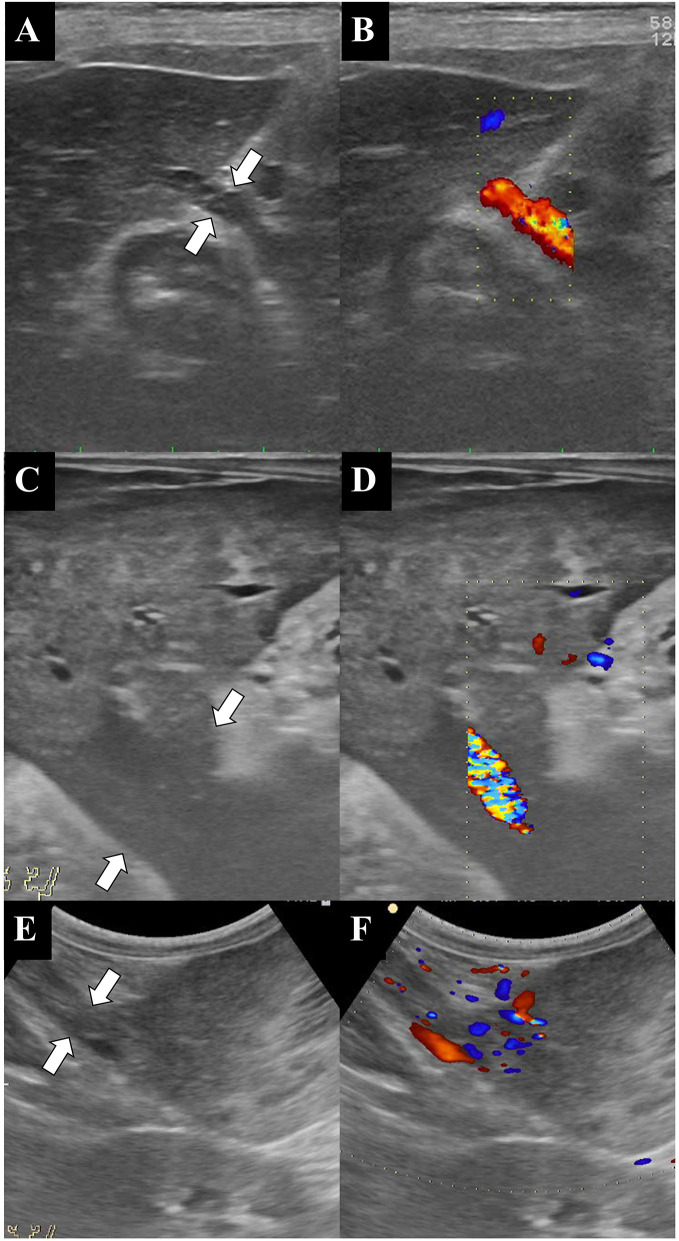
Ultrasonographic B-mode **(A,C,E)** and color Doppler **(B,D,F)** images of the liver, the pedicle, and the mass. The color Doppler images clearly show the blood supply from the liver to the mass. Note that the large abdominal masses are connected to the liver by pedicles of various sizes (white arrows). **(A,B)** Dog 2, **(C,D)** dog 4, and **(E,F)** dog 5.

Only four dogs underwent abdominal US (dogs 2, 4, 5, and 7). On US, the masses showed various architectures and echogenicities, from cystic to solid and hypoechoic to hyperechoic compared to normal hepatic parenchyma, regardless of histologic type. For all dogs that underwent US, except dog 2, the origin of the mass was confirmed during the course of the initial examination. For dog 2, US was repeated based on the CT result, and the mass was found to be connected to the liver. Three out of the four dogs that underwent US had parenchymal pedicles with hepatic parenchyma and vasculature (Figure 2). However, in dog 2, only a hypoechoic stalk with vasculature and without hepatic parenchyma was identified. On Doppler US, vascularization from the liver was confirmed for the masses in all four dogs. Regional lymphadenopathy was found in one dog (dog 2). Peri-tumoral peritonitis was found in dogs 4 and 5, with concurrent ascites in dog 4.

**Figure 3:**
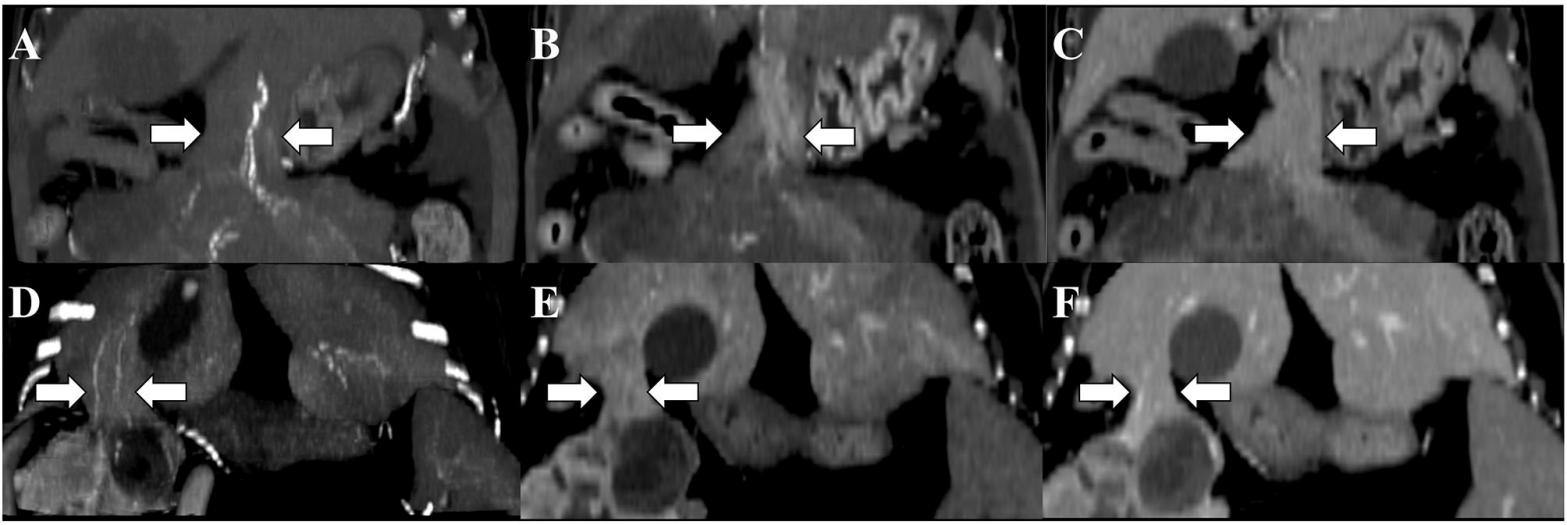
Computed tomography images of the pedunculated liver masses in the arterial **(A,D)**, portal **(B,E)**, and equilibrium **(C,F)** phases. Maximum intensity projection (MIP) was used for arterial phase imaging. Although the connectivity between the liver and the masses is determined by parenchymal pedicles (white arrows), identification of the feeding artery on the arterial phase with MIP allows confirmation of the pedicle's origin. Window level: 60; window width: 400. **(A–C)** Dog 4 and **(D–F)** dog 5.

Based on the CT scans, two types of pedicles [parenchymal (*n* = 6) and vascular (*n* = 1)] were identified. Triple-phase CT scans were performed for three dogs (dogs 4, 5, and 6), and the remaining four dogs were subjected to only pre- and post-contrast equilibrium phase scans. A feeding vessel from the hepatic artery to the mass was identified in all dogs that underwent triple-phase CT; this connection was best seen in MIP images (Figure 3). Based on the CT scans, the origins of the masses were as follows: left lateral liver lobe (*n* = 2), left medial liver lobe (*n* = 1), right medial liver lobe (*n* = 1), right lateral liver lobe (*n* = 1), caudate liver lobe (*n* = 1), and quadrate liver lobe (*n* = 1). Regional lymphadenopathy was found in five dogs (dogs 1–5), with peri-tumoral peritonitis in three (dogs 3–5) and ascites in one (dog 4). There was no evidence of metastasis to the lung.

All seven dogs underwent histopathological and cytologic examinations *via* laparotomy (*n* = 5) or fine-needle aspiration (*n* = 2, dogs 1 and 6). Three hepatic masses were identified as benign [hepatic adenoma (*n* = 1) and nodular hyperplasia (*n* = 2)], and four were identified as malignant [HCC (*n* = 3) and cholangiocarcinoma (*n* = 1)]. After surgery, all dogs recovered without any complications, and there was no evidence of recurrence or metastasis a month later.

## Discussion

Pedunculated or exophytic liver masses are masses that protrude from the liver. This report describes the multimodality imaging features of pedunculated liver masses in dogs, which are often difficult to diagnose. Based on the radiographic images, masses were found at various locations, including in the craniodorsal, cranioventral, and mid-abdominal regions. Determining the origin of a mass based on radiography alone is impossible, as there are multiple organs to consider. On ultrasonography, the masses were found to have various morphologies, ranging from cystic to solid and hypoechoic/hyperechoic to normal hepatic parenchymal. The pedicle connecting the mass and the liver was overlooked on the initial ultrasonographic examination by an experienced practitioner in one case; however, it was identified on follow-up US after CT had identified the connection, indicating that identifying the pedicle can be challenging on ultrasonographic examination. Connections between a mass and the liver were determined based on shared hepatic vasculature between the two on CT images. These findings were particularly easy to notice on arterial phase images with MIP.

Breed, sex, and body weight were randomly distributed, suggesting that they are not predisposing factors, although statistical analysis was not performed due to the small sample size. However, age seemingly acts as a predisposing factor, as all dogs in this study were in the geriatric age group. This could be due to the etiology of the neoplastic pathology of pedunculated liver masses or simply due to bias because of the small sample size (11).

All dogs except dogs 6 and 7 had elevated hepatic enzyme levels; however, this alone cannot be used to confirm or rule out hepatic neoplasia or any specific histopathologic type, as various diseases that affect the hepatobiliary system and even other organ systems can lead to such elevated profiles (12, 13). Thus, hepatic enzyme profiles should be used as a diagnostic indicator only in conjugation with the results of radiography and other modalities.

In humans, the etiology of pedunculated liver masses is currently unknown, but the most commonly accepted hypothesis is that they are associated with accessory liver lobes (10, 14), which are rare, anatomically anomalous supernumerary lobes associated with an autosomal recessive gene (14). The condition results in additional liver tissue production, which can be attached to the liver or remain completely separate from the hepatic parenchyma (called an ectopic liver) (15). Accessory liver lobes are classified as either sessile, pedunculated, or ectopic according to their anatomic relationship with the liver (10). They do not possess a complete vascular system, which can result in functional deficits and increase their susceptibility for neoplasm development (14). They are relatively small in size and can develop in all liver lobes but are most commonly found in the left lobe, known as Riedel's lobe in human medicine (10). In the present study, the pedunculated liver masses originated from all liver lobes, and there did not seem to be any lobar predisposition.

In the current study, the size of the mass and that of its pedicle did not seem to be related. Additionally, pedicle size and type did not show a clear association with tumor malignancy. One significant finding is that the vascular pedicles, including that of a massive hepatic tumor, were distinctly small and were not easily identifiable, even on CT images. In humans, the presence of a pedicle is not known to be correlated with tumor malignancy. Some studies have reported that, in comparison to typical HCCs, pedunculated HCCs are larger in size and have less vascular invasion, less metastasis, and a more favorable prognosis due to capsule formation and wide surgical margins (3); however, the results of other studies do not support these findings (16). Therefore, the peduncle is considered an anatomic feature and should not be used to predict the occurrence of a specific tumor or of malignancy. In the present study, half of the dogs had benign tumors regardless of mass size and pedicle type. In addition, in dogs with malignant tumors, including a pedunculated HCC, no evidence of metastasis was observed at short-term follow-up (1 month). Further studies are needed to determine the clinical relevance and the prognosis of the pedunculated liver mass in dogs.

Even though the presence of a peduncle is an anatomic (not pathological) feature, it does have some clinical significance. Radiologically, hepatic masses are known to protrude caudoventrally or caudodorsally in the lateral direction and to have a tendency to displace the gastric axis caudodorsally (9). However, in this study, some dogs were classified as having a mid-abdominal mass despite the hepatic origin and had gastric axis deviation in various directions. Clinically, this means that, based on radiographic examination, pedunculated liver masses can easily be mistaken for masses originating from other organs, and since radiography is one of the most routinely used screening technique in daily veterinary practice, this can result in the misdiagnosis of these masses. In addition, since it is also challenging to find the pedicle using abdominal US in most dogs, especially in the case of vascular pedicles, only one dog was correctly diagnosed initially. Furthermore, in addition to the difficulties that they pose with regard to diagnosis, it has been reported that pedicles can rotate and even become fatal. Therefore, a pedunculated liver mass, although rare, should be considered as a differential diagnosis during surgical planning and prognosis assessment if a dog shows the abovementioned radiographic characteristics.

In the present study, both US and CT examinations showed better sensitivity than radiography with regard to the diagnosis of pedunculated liver masses, although a statistical analysis was impossible due to the small sample size. All dogs that underwent ultrasonographic and CT examinations were successfully diagnosed as having a hepatic mass. However, the radiologists who participated in this study were subjectively of the opinion that CT examinations could not be replaced by US as a diagnostic imaging tool since CT examinations were more reliable than US when it came to topographic information such as the size and the location of the mass and the distribution of the vasculature for both radiologists and surgeons during the surgery planning phase. Moreover, diagnostic sensitivity depends on the scanning skill of the operator, which was also demonstrated in this case series, as the pedunculated nature of these liver masses could not be identified by the referring veterinarians on US prior to referral.

Multiplanar reconstruction was beneficial in determining the exact location of the mass and the size of the pedicle. The MIP images of the arterial phase clearly showed that hepatic vasculature had continuity with the mass and enabled the identification of the feeding artery (17). The CT characteristics of hepatic masses have been classified and well-documented based on tumor malignancy (18). The characteristics of triple-phase CT findings were identical to those of previous reports (4, 15) and of typical hepatic tumors (18).

The limitations of this study include the small sample size, which prevented us from statistically verifying the findings. In addition, because the study was retrospective, the same tests (i.e., abdominal US and triple-phase CT) were not performed in all dogs. Moreover, we could not assess the long-term survival, recurrence rate, and post-surgical complications. Finally, the study was conducted at two centers that had two different types of multi-detector CT scanners.

In conclusion, the pedunculated liver mass is an anatomic variant of the hepatic mass and should be considered as a differential diagnosis for a mass located cranial to the mid-abdomen, even if it is separated from the liver on radiographic images. These masses can easily be misdiagnosed, but our findings indicate that CT is a useful tool for diagnosis, evaluation, and surgical planning in dogs suspected to have pedunculated liver masses.

## Data Availability Statement

The original contributions presented in the study are included in the article/Supplementary Materials, further inquiries can be directed to the corresponding author.

## Ethics Statement

Ethical review and approval was not required for the animal study because as a case report, this study does not require ethical review and approval. Instead, all owners of enrolled patients were informed about study and agreed to provide medical record of their dogs. Written informed consent was obtained from the owners for the participation of their animals in this study.

## Author Contributions

JKo and JH contributed to abdominal ultrasonography scanning, image interpretation, and manuscript writing. HY and KE contributed to CT scanning, image interpretation, and manuscript editing. JKi contributed to the study design, abdominal ultrasonography scanning, CT scanning, image interpretation, confirmation of radiological diagnoses, and manuscript editing and correction. All authors contributed to the article and approved the submitted version.

## Conflict of Interest

The authors declare that the research was conducted in the absence of any commercial or financial relationships that could be construed as a potential conflict of interest.
