# Supplementary material for: Case Report: Multimodal Imaging Features of Pedunculated Liver Masses in Seven Dogs
Source: Front Vet Sci. 2020 Nov 24;7:581922. doi: 10.3389/fvets.2020.581922 (PMC7732477; doi:10.3389/fvets.2020.581922)
Supplement: Supplementary file 1 [file Table_1.docx]

| **Case** | **Breed** | **Age (years)** | **Sex** | **BW (kg)** | **Chief complaint** |
| --- | --- | --- | --- | --- | --- |
| 1 | Cocker Spaniel | 9 | MC | 9 | Abdominal mass |
| 2 | Maltese | 12 | FS | 3.7 | Abdominal mass |
| 3 | Shih-Tzu | 14 | FS | 4.8 | Abdominal mass |
| 4 | Shih-Tzu | 13 | MC | 5.6 | Hepatic mass |
| 5 | Schnauzer | 10 | FS | 7.1 | Skin nodule |
| 6 | Schnauzer | 12 | MC | 8.1 | Splenic mass |
| 7 | Cocker Spaniel | 14 | FS | 11.3 | Pancreatic mass |

**Supplementary Table 1.** Summary of breed, age, sex, body weight, and chief complaint in 7 dogs with pedunculated liver mass
BW, body weight; MC, male castrated; FS, female spayed female
